# Effects of national health benefits expansion policy on out-of-pocket payments and utilization of patients with four major catastrophic diseases

**DOI:** 10.12688/healthopenres.13475.1

**Published:** 2024-02-01

**Authors:** Minjeong Kim, Jangho Yoon, Chunhuei Chi

**Affiliations:** 1 School of Social and Behavioral Sciences, College of Public Health and Human Sciences, Health Management and Policy, Oregon State University, Corvallis, Oregon, USA; 2 Division of Health Services Administration, Department of Preventive Medicine & Biostatistics, F. Edward Hebert School of Medicine, Uniformed Services University, Bethesda, Maryland, 20814, USA

**Keywords:** National Health Insurance, Universal Health Care, Benefits Coverage, Benefits Expansion Policy, High-cost procedures, Health Accessibility, Major Catastrophic Diseases

## Abstract

**Background:**

As South Korea achieved universal health care (UHC) in 1989, patients’ access to low-cost health services has highly increased. However, as liability for high-cost procedures is still high, patients’ accessibility to high-cost services is has been limited. For this reason, the Korean government has implemented an initiative of the “Mid-term Health Benefits Security Plan” to expand the health benefits coverage since 2005. Nevertheless, it has been criticized as the policy has yet to show any transparent evidence of reducing patients’ out-of-pocket costs since its implementation. This study aims to identify if the benefit expansion policy affected a reduction of patients’ health care spending and utilization after policy implementation.

**Methods:**

We analyze data from the Korean Health Panel Survey for years 2009-2016, a nationally representative survey of non-institutionalized Korean citizens that provide the most comprehensive information on health care utilization and costs. We utilize two-part difference-in-differences (DID) models to estimate the patients' probability of accessing any care and the intensity of care, health care spending and utilization, conditional on the initiated care

**Results:**

The total out-of-pocket(OOP) payments and inpatient spending decreased by USD 175.33 (p = 0.033) and USD 358.86 (p =0.018), respectively, which were statistically significant. Outpatient spending increased by USD 57.43 (p =0.607), but it was not statistically significantly associated with the policy implementation. In utilization, there were no significant changes in either the number of visits or hospital stays.

**Conclusions:**

Even though we found that the policy led to a reduction in patients' OOP spending, the effects of the policy were largely limited to inpatient services and patients with high incomes. As the limited benefits of the policy to the particular services and patients might raise some equity issues, the government needs to extend the range of coverage more broadly so that a more comprehensive population can benefit from the policy.

## Introduction

Since 1989, when the South Korean government achieved universal health coverage (UHC), 97.1% of the population has been covered under the national health insurance (NHI) system with remaining 3% receiving services through the Medical Aid Program ( Kwon, 2015; MOH, 2020). Despite the complete population coverage, patients who require high-cost procedures still experience catastrophic costs due to the short range of covered services in a national package.

Health insurance schemes consist of three interrelated components: population coverage, a range of services covered, and the extent of financial protection from costs ( OECD, 2016). Even though population coverage is fulfilled, if the range of the covered services is insufficient, patients can still encounter a risk of paying catastrophic costs of care.

In fact, South Korea displays a lower rate of nationally covered services in a package out of all available healthcare services than other Organization for Economic Co-operation and Development (OECD) countries. While the Korean NHI covers 61% of health service expenditure, the other 32 OECD countries publicly covered, on average, 74% of the services expenditure in 2019. In the case of the top 10 countries, the governments include above 80% of the healthcare services in a package ( OECD, 2021).

This shortage of covered services results in relatively high out-of-pocket (OOP) payments for patients for care. In 38 OCED countries, 20% of health spending, on average, is financed by patients’ OOP payments, but in South Korea, 30% of health spending is financed by OOP payments ( OECD, 2021).

The NHI’s limited range of covered services stemmed from the South Korean government’s fundamental principle of the “low contributions, limited benefits, and high cost-sharing” policy to initially increase population coverage. However, since 2005, the government has implemented the “Mid-term Health Benefits Security Plan” to compensate for the lack of a range of covered services ( Son, 2015). With the expanded nationally covered services, the government aimed to reduce patients' OOP payments by providing covered services at lowered costs through fee scheduling ( Son, 2015).

Despite the government's expectations, however, the policy has been criticized as it has yet to show any noticeable intended result. Previous studies presented that the policy did not lead to a reduction of patients' OOP payments ( Cho *et al.*, 2016; Choi & Jeong, 2012; Choi *et al.*, 2014; Kim & Kwon, 2015; Kim & Shin, 2017; Kim *et al.*, 2014 ; Lee, 2018; Park & Jung, 2019). These results might arise from the fact that the previous studies evaluated the policy in different phases of the “Mid-term Health Benefits Security Plan” over three phases (First: 2005–2008, Second: 2009–2013, Third: 2014–2018). Depending on which phase of the initiative they examined, the assessment can be different.

Also, the indistinct results might result from the fact that they did not carefully consider the features of health claims data. The health claims data have features of high skewness with a large mass at zero and heterogeneity between zero- and nonzero- populations ( Deb *et al.*, 2017). As a small portion of the population takes up a substantial portion of total health expenditures, most health claims data display a distribution of considerable skewness with a high peak at zero ( Deb *et al.*, 2017). Heterogeneity between zero- and non-zero values in health claims data also needs close attention because the heterogeneity between the populations can have significant political implications ( Deb *et al.*, 2017). In healthcare services, once care is initiated by patients, physicians can heavily influence the intensity of the subsequent care in terms of quantity, quality, and costs of care ( Newhouse & Phelps, 1976). In this case, the distinction between the analyses of the probability of accessing care and the intensity of the care conditional on the initiated care is required to take into account the heterogeneity ( Ringel *et al.*, 2002). If these features of health claims data are neglected, the estimates can be biased ( Deb *et al.*, 2017).

In this study, we tried to obtain empirically accurate estimates to verify the policy’s efficacy on patients’ health expenditure and utilization by employing appropriate analytical approaches to consider the features of the health claims data, such as Two-Part model and generalized linear model (GLM) estimation.

Furthermore, we attempt to highlight the significance of a sufficient range of covered services in addition to population coverage to prevent patients from paying catastrophic costs for care.

## Methods

### Data source & sample

Data from the Korea Health Panel Study (KHPS), which have conjointly been conducted by Korean Institute for Health and Social Affairs (KIHSA) and National Health Insurance Service (NHIS) in South Korea, is used for this study (Version 1.6). KHPS has been designed to generate representative data on the population’s expenditure and utilization of healthcare services and in/direct factors affecting the expenditure and utilization in South Korea since 2008. Hence, the survey provides in-depth data not only on patients’ annual utilization and expenditure for healthcare but also on the population's demographics, socioeconomic, and health status and behaviors ( "KHPS”, 2019).

In this study, a total of 9427 patients (28,421 observations) are examined for eight years (2009 to 2012 [pre], and 2013 to 2016 [post]), consisting of 3211 patients (8504 observations) in a policy group (34.1%) and 6216 patients (19,917 observations) in a control group (65.9%). We excluded the Medicaid beneficiaries to examine exclusively the national health insurance enrollees.

### Variable

As the main outcome variable, patients' OOP payments (cost-sharing for covered services plus full costs for non-covered services) are measured as the total OOP payments and inpatient and outpatient payments, respectively. In the data set, the OOP payments are recorded as Korean currency, won (₩), and the KRW (Korean Currency) was converted to USD at a rate of USD 1= KRW 1311. For the second outcome variable, the change in patients' utilization is measured as the number of visits for outpatient services and hospital days for inpatient services.

The main explanatory variables consist of three variables: a policy group, a control group, and policy × post. The policy group is assigned if individuals were diagnosed with one of the four major catastrophic diseases (cancers, cardiovascular-, cerebrovascular-, and rare diseases) designated in the 'Government's guideline of health benefits expansion initiative for critical and rare illnesses' ( MOH, 2013). We matched the names of diagnoses in the guideline and in the data set to sort out the policy group.

From 2012, the codes of diagnoses were recorded by the KCD-6 (Korean standard classification of disease and cause of death) system, consisting of 3–5 digits. However, since the codes were provided only three digits out of 3–5 digits in the data set, we assigned the policy group as long as the three digits of the codes were matched with the government’s guideline.

From 2009 to 2011, the codes were recorded by four digit- (2009) and five digit-numbers (2010–2011). For 2009, like in 2012, we assigned the policy group as long as four digits-numbers were matched. For 2010–2011, however, we matched all five digits-number as the data set offers all five digit-numbers of the codes.

For the control group, we referred to the government’s report ‘2012 Annual Survey on Medical Expense of National Health Insurance Enrollees ( NHIC, 2013)’. This report lists the top 50 diseases causing high medical expenses per person in 2012. Among the 50 diseases, we selected 12 diseases after excluding major diseases in the policy group. We added five more diseases from the list in the ‘Analysis on Patients of High-Paying and Critical Diseases among NHI Enrollees (2005)’ ( NHIC, 2007) to match the control group compared with the policy group in terms of annual medical spending. As a result, 17 diseases are selected for the control group ( Table 1).

**Table 1.** 17 diseases selected for the control group.

No.	KCD-6 Disease Codes	Diagnoses	Reference
1	A41	septicemic	Annual Survey on Medical Expenses of National Health Insurance Enrollees ( NHIC, 2013)
2	A55-56	Sexually transmitted diseases due to Chlamydia trachomatis	
3	A86	Viral meningoencephalitis NOS	
4	B20-B24	Human immunodeficiency virus (HIV) disease	
5	B05	measles	
6	F03	Unspecified dementia	
7	F10	Mental and behavioral disorders due to the harmful use of alcohol	
8	G80-G83	Cerebral palsy and other paralytic syndromes	
9	N17, N19	Renal failure (N18 excluded)	
10	P05-P07	Disorders related length of gestation and fetal growth	
11	P28	Primary failure to expand terminal respiratory units	
12	S72	Fracture of femur	
13	M51	Other intervertebral disc disorders	Analysis of Patients of High-Paying and Critical Diseases among NHI Enrollees ( NHIC, 2007)
14	M48	Other spondylopathies	
15	M17	Gonarthrosis [arthrosis of knee]	
16	M25	Senile cataract	
17	E11	Non-insulin-dependent diabetes mellitus	

* To compose a control group for the study, we primarily referred to the government's report '2012 Annual Survey on Medical Expense of National Health Insurance Enrollees'. We added five more diseases from the 'Analysis on Patients of High-Paying and Critical Diseases among the NHI Enrollees (2005)' to make the control group comparable with the policy group in terms of an annual medical spending.

For the period variables, the years 2009–2012 are set as the pre-policy period, and the years 2013–2016 are set as the post-period period. The 'Mid-term Health Benefits Security Plan' was gradually planned over three-phase periods: the first (2005–2008), the second (2009–2013), and the third (2014–2018) ( Son, 2015).

In 2013, as the president Park Gun-Hye took office, she underscored a reduction of a range of non-covered services to diminish patients’ financial burdens, especially for patients with catastrophic diseases. In contrast to the fact that the government primarily focused on reducing a coinsurance rate of covered services in the second phase (from 20% to 10% in 2005 and from 10% to 5% in 2009), the Park Gun-Hye administration attempted to reduce a portion of non-covered services in a package by switching the non-covered services to the nationally covered services since 2013. As the new President, Moon Jae-in, was elected in 2017, President Park Geun-Hye’s initiative was completed in 2016. Thus, we set 2009–2012 as the pre-period and 2013–2016 as the post-period.

We also examine the effects of the benefits expansion policy by patients’ income levels with the Triple Differences (TD). As the additional comparison variable, the income level is categorized by four different income groups, from high to low, specifying hhinc_c = 0 as the highest and hhinc_c = 3 as the lowest income level.

To control for other factors that might affect the outcome, covariates are included based on Andersen’s health behavior model ( Andersen & Newman, 2005). In theory, Andersen and Newman indicate ‘Predisposing’, ‘Enabling’, and ‘Illness Level’ as individual determinants of health services utilization. For the ‘Predisposing’ component, we include demographic factors (age, sex, educational level, marital status, the number of children, and residence areas) in this study. For the ‘Enabling’ component, economic factors (total annual house income and status of employment) and private health insurance (PHI) factors (the number of PHI and total monthly private premium) are included. For the ‘Illness level’ components, health status factors (the number of chronic diseases, self-assessed health status, status of disability) are included.

### Empirical strategy

***Difference-in-Differences (DID) approach*.** We apply the DID approach to identify causal effects between the benefits expansion policy and patients’ health expenditure and utilization while controlling for unobservable confounding factors. With the standard DID format, we compare the pre-post outcomes of the policy and the control group ( Callaway & Sant'Anna, 2018; Dimick & Ryan, 2014).

The DID estimation is only validated under the common parallel condition in pre-periods. Thus, we inspect whether the policy and the control group have a parallel time trend in pre-periods by multiplying a policy variable with a year variable (policy x year) in pre-policy periods. As shown in Table 2, the five models (three out-of-pocket payments and two utilizations models) with covariates display statistical insignificance at all significant levels. For this reason, we can assume that there are no other unobserved factors that might affect to estimate the policy’s effects through the DID estimation.

**Table 2.** Common parallel assumption tests.

Models	P-value (policy × year [pre-periods])	
	W/O covariates	W/ covariates
**OOPP**		
1. Total OOP *	0.072	0.248
2. Inpatient OOP	0.387	0.194
3. Outpatient OOP	0.000	0.378
**Utilities**		
4. Visits to hospitals	0.005	0.788
5. Stays in hospitals	0.092	0.430

* OOP: Out-of-pocket ** The common parallel assumption tests were implemented to examine validity of using the Difference-in-Differences analysis. As the coefficients of the five models are all statistically insignificant in pre-policy periods [2009–201], it is suggested that there might not be other unobserved factors that can affect to estimate the policy effects.

The DID approach can be more convincing when adding more comparison groups and differencing the effects of the groups out at different time periods in the way of difference-in-difference-in-differences (Triple Differences, TD). More specific effects of the policy can be identified by using the Triple Differences ( Wing *et al.*, 2018).

The benefits expansion policy can differently affect the patients depending on which income level the patients are involved in. Patients with lower incomes can be more sensitive to the policy's effects due to a higher possibility of encountering astronomical costs for care. Therefore, examining how the policy affects the patients according to their income levels can be worthwhile to evaluate the policy with TD estimation.

First, the two DID models are specified in equation (1) and equation (2) for the health expenditure and utilization response variables.

$${\text{OOPP}}_{it}=\beta_{0}+\beta_{1}{policy}_{i}+\beta_{2}post_{t}+\beta_{3}policy_{i}\times post_{t}+\beta_{4}x_{it}^{\prime }\beta +\varepsilon_{\mathrm{it}}\;(1)$$

$${\text{Utilization}}_{it}=\beta_{0}+\beta_{1}policy_{i}+\beta_{2}post_{t}+\beta_{3}policy_{i}\times post_{t}+\beta_{4}x_{it}^{\prime }\beta +\varepsilon_{\mathrm{it}}\;(2)$$

where OOPP _*it*_ indicates patients' out-of-pocket payments for inpatient and outpatient services, and total OOP payments of individuals *i* during t year in an equation (1). *policy _i_* is the patients affected by the policy implementation; therefore, 1 if patients have been diagnosed with the four major catastrophic diseases (cancers, cardiovascular-, cerebrovascular-, and rare-diseases), and 0 otherwise. *post _t_* is the post-period of the policy implementation (2013–2016), so 1 if the period is included in 2013–2016, 0 otherwise. *policy _i_* × *post _t_* is the interaction term of which the coefficient ( *β*_3_) captures the average treatment effects of the benefits expansion policy on policy group's total OOP payments, and OOP payments for inpatient and outpatient services.

In equation (2), Utilization _*it*_ denotes the patients' number of visits for outpatient services and hospital days for inpatient services. Like equation (1), the coefficient of the interaction term ( *β*_3_) captures the average treatment effects of the policy on the policy group’s healthcare utilization.

To examine the effects of the benefits expansion policy depending on patients’ income levels, the TD model is specified in equation (3) below.

$$\begin{array}{l} OOPP_{it}=\beta_{0}+\beta_{1}post_{t}+\beta_{2}policy_{i}+\beta_{3}income_{i}+\beta_{4}post_{t}\times policy_{i}\\ \;+\beta_{5}post_{t}\times income_{i}+\beta_{6}policy_{i}\times income_{i}+\beta_{7}policy_{i}\times income_{i}\times post_{t}\\ \;+\beta_{8}x_{it}^{\prime }\beta +\varepsilon_{it} \end{array}\;(3)$$

The patients’ income level in the TD model is categorized into four groups, from high to low. Thus, *income _i_* =0 if patients are included in the highest level and *income _i_* =3 in the lowest level. The coefficient of the triple interaction term ( *β*_7_) captures the different effects of the policy depending on patients’ income levels. Patients with a lower income level are assumed to be more significantly affected by the government’s benefits expansion policy than those with a higher income level.

A vector of observed characteristics of observations includes the social demographic factors (age, sex, education level, marital status, the number of children, residence areas), the economic factors (a status of employment), the health status factors (the number of chronic diseases, self-assessed health status, and status of disability), and the private health insurance factors (the number of PHI possessed by patients and total monthly private premium). *ε _it_* is an error term.

***Two-Part Model*.** With the Two-Part model, we divided the DID model into two separate estimations, consisting of the patients’ probability of receiving care (zero- *versus*-non zero) in the first part and the intensity of care, health spending and utilization, in the second part, conditional on the initiated care in the first part.

In healthcare services, whether seeking care or not is solely patients’ choice. However, once care is initiated by patients, physicians’ decisions heavily impact the intensity of care ( Newhouse & Phelps, 1976). In such cases, the semi-continuous estimation, like the Two-Part model, needs to be applied to consider the heterogeneity between the patients with or without the first care. As the two-part model provides an appropriate approach to account for the skewness and heterogeneity of health claims data, we can obtain accurate estimates ( Folland *et al.*, 2013; Ringel *et al.*, 2002).

***Estimation*.** We use the GLM to estimate the first and second parts of the two-part model. The GLM allows to specify of various link functions and distribution families for all types of the annual panel data ( Deb *et al.*, 2017; Gad & Kholy, 2012).

In the first part of the two-part model, the ‘identify’ link function and the ‘gaussian’ distribution family are applied to estimate the probability of observing a zero and non-zero outcome. For the second part, box-cox tests (for a link function) and modified park tests (for a distribution family) are implemented to determine which link function and distribution family are appropriate to fit in the continuous response variables.

The Health Expenditure Model

$$E(oopp_{it}|x_{it})=[\begin{array}{ll} \text{E}\{\text{Pr}(oopp_{it}=1|x_{it})\}={x_{it}}^{\prime }\beta , & oopp_{it}\sim Gaussian\;(4)\\ \text{sqrt}\{E(oopp_{it}|oopp_{it}>0,x_{it})\}={x_{it}}^{\prime }\beta , & oopp_{it}\sim Gaussian\;(5) \end{array}$$

In equation (4) and equation (5), *oopp _it_* represents patients' total OOP payments and the OOP payments for inpatient and outpatient services.

The ‘square root’ link function and the ‘gaussian’ distribution family are employed to the expenditure response variable after the box-cox and the modified park tests.

A vector of observable characteristics of the population includes the social demographic, economic, health status, and behavioral factors.

The Patients' Utilization Model

$$E({\text{utilization}}_{it}|x_{it})=[\begin{array}{ll} \text{E}\{\text{Pr}({\text{utilization}}_{it}=1|x_{it})\}={x_{it}}^{\prime }\beta & utilization_{it}\sim Gaussian\;(6)\\ \text{log}({\text{utilization}}_{it}|{\text{utilization}}_{it}>0,x_{it})={x_{it}}^{\prime }\beta & utilization_{it}\sim NB\;(7) \end{array}$$

In equations of ( 6) and ( 7), *utilization _it_* indicates patients' numbers of visits for outpatient services and hospital days for inpatient services.

For the utilization response variable in the second part, we fit the ‘log’ link function and the ‘negative binomial’ distribution family (NB) after the box-cox tests and the modified park tests. Since the response variables are the count variables, we fit the NB estimation instead of Poisson to release the equidispersion property of the Poisson estimation. With the NB estimation, we can appropriately adjust the overdispersion of the standard errors.

A vector of observable characteristics of observations includes social demographic factors, economic factors, health status factors, and private health insurance factors.

We also control for the within-subject correlation arising from using the repeated data by manually creating the time-demeaned data. We compare the estimates of an original GLM with those of a GLM with time-demeaned data to identify potentially omitted time-invariant individual effects.

For the data analysis, STATA version 15.1 is used.

## Results

With regard to the survey population, the control group is quite comparable to the policy group as seen in Table 3 on social demographic factors (age and gender), economic and health status factors, and outpatient spending and utilization. However, spending and utilization of the policy group for inpatient services are inherently higher than the control group due to the severity of the diseases.

**Table 3.** Description of the survey population [pre: 09–12, post: 13–16].

Variables	Definitions	Policy (n=3211, 34.06%)		Control (n=6216, 65.94%)	
		Pre (n=2113)	Post (n=2016)	Pre (n=3932)	Post (n=4790)
**Outcomes**					
< Out-of-pocket payments>					
Outpatient services		$505.98 (711.24)	$531.23 (791.54)	$387.79 (620.62)	$473.09 (675.12)
Inpatient services		$539.86 (1323.85)	$646.34 (1732.21)	$283.13 (1008.80)	$354.29 (1148.35)
Non-covered services		$299.55 (915.02)	$595.05 (1259.93)	$148.78 (582.34)	$412.65 (921.87)
Total out-of-pocket payments		$1,047.16 (1590.94)	$1,176.67 (1971.71)	$672.05 (1239.75)	$827.80 (1401.42)
< Utilizations >					
Number of visits		30.6(32.5)	34.7(34.8)	35.4(33.8)	39.2(36.7)
Lengths of hospitalization		7.6(23.4)	9.4(31.9)	4.9(19.9)	5.2(21.2)
**Explanatory variables**					
< Social demographic factors >					
Age	the average age of the population	60.0(14.5)	66.8(12.3)	64.1(12.2)	66.7(12.2)
Female	=1 if female; 0 otherwise	62.1%	50.9%	62.4%	62.3%
Education (Reference: < High school)					
High school	=1 if a high school graduate	47.0%	41.0%	32.0%	35.0%
College	=1 if have college or above	16.5%	14.5%	10.6%	10.2%
Marital status	=1 if married; 0 otherwise	80.2%	77.6%	74.4%	71.8%
Children	An average number of children	0.96(1.0)	0.67(1.0)	0.78(1.1)	0.65(1.0)
Residence	=1 if living in Seoul (Capital)	15.0%	12.3%	13.2%	11.3%
<Economic factors>					
House income	Average yearly household income (in ₩10,000)	$27,484.5 (24,870.3)	$27,492.8 (15,895.5)	$22,960.3 (14,472.9)	$25,329.5 (14,321.1)
Individual income	Average yearly individual income (in ₩10,000)	$13,924.4 (13,131.2)	$14,579.7 (15,895.5)	$12,595.7 (14,472.9)	$13,088.5 (14,321.1)
Employment	=1 if currently employed	43.8%	40.5%	48.6%	46.4%
Medical expense	Average individual medical expense	$1265.7 (1726.3)	$1472.0 (2190.5)	$883.3 (1339.8)	$1057.6 (1527.3)
< Health status factors >					
Chronic diseases	Number of chronic diseases	3.7(2.4)	8.5(3.0)	3.9(2.3)	8.3(3.0)
Perceived health status (PHS) (Reference: Very bad)					
Good	=1 if PHS is very good or good	17.4%	17.0%	20.0%	21.7%
Fair	=1 if PHS is fair	26.8%	40.2%	28.5%	42.4%
Bad	=1 if PHS is very bad or bad	24.7%	35.5%	21.4%	31.5%
< Private health insurance (PHI) >					
No. of PHI	Number of PHI possessed	1.1(1.4)	1.0(1.4)	0.9(1.2)	1.1(.14)
PHI premium	Total monthly private premium	$47.54 (77.56)	$43.67 (76.67)	$40.20 (78.64)	$47.29 (77.17)

* Standard errors in parentheses KRW (Korean Currency) is converted to USD at a rate of $1= ₩ 1,311.00 (2022.08)

### Out-of-pocket payments

***Total out-of-pocket payments*.** For the first part of the GLM model, the coefficient on the interaction term (policy × post) displays − 0.0052 (p =0.137), which implies that the probability of spending total OOP payments of the policy group decreases after policy compared to the control group even if it is insignificantly associated with the policy. In the second part, conditional on patients with any spending for OOP payment, the coefficient on the interaction term (policy × post) shows − 144.37 (p =0.000), suggesting that the policy group’s total amount of OOP payments decrease after policy compared to the control group, which is statistically significantly associated with the policy ( Table 4).

**Table 4.** Coefficients from the DID model for total OOP payments.

	GLM		GLM(w/time-demeaned data)	
	First part	Second part	First part	Second part
Policy	0.0005 (0.0022)	163.8454 *** (17.8148)	0.0028 (0.0030)	122.5281 ** (47.3257)
Post	-0.0004 (0.0043)	101.2048 (59.5705)	0.0058 (0.0038)	199.1843 * (81.5759)
Policy#Post	-0.0052 (0.0035)	-144.3704 *** (33.4242)	-0.0063 * (0.0031)	-120.0654 * (56.2957)
Age	-0.0000 (0.0001)	-2.4537 (1.6850)	-0.0012 (0.0009)	-71.0348 ** (24.2592)
Female	0.0027 (0.0020)	-37.5377 (31.7370)	0.0000 (.)	0.0000 (.)
Edu_high school	-0.0023 (0.0020)	-59.7178 (33.1184)	-0.0011 (0.0016)	-27.5206 (268.4856)
Edu_College	-0.0006 (0.0032)	54.1600 (29.9736)	-0.0000 (0.0013)	-13.3602 (208.1130)
Married	0.0058 * (0.0027)	45.1038 (49.4015)	0.0074 (0.0076)	-101.2566 (51.9422)
Children	0.0005 (0.0011)	-17.7270 (9.8013)	0.0007 (0.0017)	-18.2864 (18.6357)
Seoul	-0.0009 (0.0032)	2.0957 (19.6628)	0.0037 (0.0060)	223.0927 ** (76.8764)
House income	0.0000 (0.0000)	0.0050 (0.0028)	0.0000 (0.0000)	0.0103 (0.0070)
Employed	-0.0023 (0.0021)	1.7308 (51.3909)	0.0008 (0.0020)	-1.7737 (26.9183)
Medical Expense	0.0000 *** (0.0000)	0.0002 *** (0.0000)	0.0000 *** (0.0000)	0.0002 *** (0.0000)
Individual Income	-0.0000 (0.0000)	-0.0087 (0.0056)	-0.0000 (0.0000)	-0.0172 (0.0268)
Number of Chronic Diseases	0.0005 (0.0004)	-0.3872 (5.6565)	-0.0001 (0.0005)	3.5925 (9.7820)
Perceived Health_Very good	0.0006 (0.0049)	-69.1833 (40.8752)	0.0050 (0.0072)	8.7975 (49.8613)
Good	-0.0014 (0.0019)	-60.0229 (58.9509)	-0.0021 (0.0018)	60.4673 (35.4019)
Bad	0.0001 (0.0017)	52.1058 * (24.4902)	0.0035 (0.0023)	53.4676 (44.9474)
Very Bad	-0.0039 (0.0068)	99.0177 * (49.0825)	-0.0030 (0.0086)	-6.8853 (125.5780)
BMI	0.0001 (0.0002)	-2.6756 (2.2735)	0.0003 (0.0003)	-2.5003 (2.8280)
Disabled	0.0029 (0.0022)	-3.7413 (38.3601)	0.0001 (0.0090)	-11.8372 (85.9162)
PHI(Private Health Insurance)	-0.0007 (0.0026)	36.2755 (33.4506)	-0.0037 (0.0030)	37.0582 (42.5812)
Total_Premium for PHI	-0.0000 (0.0000)	0.0001 (0.0001)	-0.0000 (0.0000)	0.0003 (0.0002)
Number of PHI	0.0013 * (0.0006)	-10.3146 (11.9273)	0.0029 (0.0019)	-23.3786 (23.4962)
2010.year	0.0009 (0.0029)	14.3843 (25.5880)	0.0013 (0.0025)	-14.7837 (36.6358)
2011.year	0.0035 (0.0025)	49.8311 (26.0357)	0.0043 * (0.0020)	84.0161 * (40.2735)
2012.year	0.0016 (0.0028)	62.4051 * (26.2496)	0.0037 (0.0026)	116.8874 (61.5924)
2013.year	0.0007 (0.0036)	59.1434 (61.5748)	-0.0017 (0.0027)	70.7281 (56.0293)
2014.year	-0.0004 (0.0027)	-5.9703 (54.3809)	0.0020 (0.0017)	24.7574 (37.7018)
2015.year	0.0020 (0.0024)	-3.2784 (64.6775)	0.0034 (0.0018)	22.6057 (62.6857)
2016.year	0.0000 (.)	0.0000 (.)	0.0023 (0.0017)	205.9318 ** (68.6888)
_cons	0.9881 *** (0.0094)	875.9992 *** (140.6632)	-0.0023 (0.0015)	652.6380 *** (30.6938)
*N*	9986	9936	9986	3448

DID: Difference-in-Differences, OOP: Out-of-pocket, GLM: Generalized linear model Standard errors in parentheses ^*^ *p* <0.05, ^**^ *p* <0.01, ^***^ *p* <0.001 'seoul' = a reference group of patients' residency

With time-demeaned data to control for unobserved individuals’ heterogeneity, both coefficients on the interaction terms (policy × post) in the first and second parts are still negative, but they become statistically significantly associated with the policy, displaying − 0.063 (p =0.040) in the first and − 120.07 (p =0.033) in the second part. By eliminating the unobserved individual’s heterogeneity, the probability of spending the amount of total OOP payments become statistically significantly associated with the policy. Further, the estimate substantial size effect suggests the policy effect is also practically significant.

As the marginal effect of the interaction term (policy × post) displays − 229,683.8, it is suggested that the total OOP payments of the policy group decrease by USD 175.33 after the policy, which appears to be both statistically and practically associated with the policy implementation.

***Out-of-pocket payments for inpatient services*.** For inpatient services, the coefficient on the interaction term (policy × post) shows 0.0101 (p =0.571) in the first part ( Table 5), indicating that the probability of spending OOP costs for inpatient services increases compared to the control group even if it is statistically insignificantly associated with the policy. In the second part, conditional on patients with any inpatient service spending, the coefficient on the interaction term (policy × post) indicates − 3.6593 (p =0.063). The policy group’s amount of inpatient OOP payments decreased after the policy compared to the control group, which appears statistically insignificantly associated with the policy. Even though the probability of the policy group spending inpatient expenditure increases, among the patients with positive spending, the total amount of the inpatient OOP payments decreases after the policy.

**Table 5.** Coefficients from the DID model for inpatient OOP payments.

	GLM		GLM(w/time-demeaned data)	
	First part	Second part	First part	Second part
Policy	0.0892 *** (0.0142)	2.4988 * (1.2022)	0.1366 *** (0.0222)	0.9771 (2.6657)
Post	0.0555 * (0.0217)	4.0174 (2.8593)	0.0317 (0.0217)	3.1519 (8.9135)
Policy#Post	0.0101 (0.0178)	-3.6593 (1.9703)	-0.0153 (0.0222)	-7.4494 * (3.1430)
Age	-0.0003 (0.0005)	-0.1225 (0.0913)	-0.0023 (0.0052)	-2.0101 (1.7327)
Female	-0.0200 * (0.0096)	-2.5056 (1.6441)	0.0000 (.)	0.0000 (.)
Edu_high school	0.0030 (0.0099)	-1.5592 (1.5881)	0.0908 (0.0973)	-3.9787 (11.7253)
Edu_College	-0.0185 (0.0121)	3.0521 * (1.4509)	-0.0056 (0.0729)	4.6577 (21.9824)
Married	-0.0082 (0.0110)	2.1863 (2.7726)	0.0148 (0.0441)	40.5851 ** (12.7584)
Children	0.0027 (0.0045)	-0.6478 (0.5728)	0.0113 (0.0116)	-0.1008 (1.8000)
Seoul	-0.0492 *** (0.0111)	1.5543 (1.4640)	0.1463 ** (0.0463)	4.9356 (7.0400)
House income	-0.0000 *** (0.0000)	0.0001 (0.0003)	-0.0000 ** (0.0000)	0.0017 ** (0.0005)
Employed	-0.0248 (0.0133)	-1.4090 (2.0406)	0.0104 (0.0129)	-1.5777 (1.7649)
Medical Expense	0.0000 *** (0.0000)	0.0000 *** (0.0000)	0.0000 *** (0.0000)	0.0000 *** (0.0000)
Individual Income	-0.0000 (0.0000)	-0.0002 (0.0004)	0.0000 (0.0000)	0.0021 * (0.0009)
Number of Chronic Disease	-0.0057 * (0.0023)	-1.0285 *** (0.2830)	-0.0038 (0.0032)	-0.1140 (0.5587)
Perceived Health _Very good	0.0052 (0.0246)	-2.6637 (3.5772)	0.0210 (0.0281)	0.1665 (5.4823)
Good	0.0079 (0.0094)	-5.3774 (4.2129)	0.0019 (0.0109)	-1.4238 (2.6570)
Bad	0.0313 ** (0.0111)	0.1538 (1.2257)	0.0199 (0.0129)	4.3798 * (2.0461)
Very Bad	0.0700 * (0.0353)	4.2379 * (2.0781)	0.0613 (0.0390)	1.3568 (4.5030)
BMI	-0.0032 * (0.0015)	-0.0616 (0.0919)	-0.0047 ** (0.0016)	-0.1221 (0.2233)
Disabled	0.0233 (0.0161)	0.0286 (2.3534)	-0.0500 (0.0377)	-9.1621 (5.1404)
PHI (Private Health Insurance)	0.0141 (0.0121)	-0.7418 (1.9570)	0.0414 (0.0266)	-3.6416 (4.4892)
Total_Premium for PHI	0.0000 (0.0000)	0.0000 (0.0000)	0.0000 (0.0000)	-0.0000 (0.0000)
Number of PHI	-0.0054 (0.0041)	-0.2533 (0.5718)	-0.0238 * (0.0116)	-0.3899 (2.2837)
2010.year	0.0051 (0.0147)	-0.0852 (1.8373)	0.0050 (0.0124)	-0.9593 (2.6024)
2011.year	-0.0026 (0.0148)	0.1317 (1.6784)	0.0010 (0.0116)	1.1804 (3.4838)
2012.year	0.0141 (0.0160)	1.1273 (1.6320)	0.0038 (0.0132)	1.4380 (5.2496)
2013.year	-0.0311 (0.0199)	-1.0433 (3.1307)	0.0112 (0.0154)	2.8630 (4.2029)
2014.year	-0.0191 (0.0139)	2.7915 (2.8264)	0.0028 (0.0100)	3.0686 (2.8687)
2015.year	-0.0122 (0.0145)	3.0930 (2.8919)	0.0102 (0.0101)	4.5568 (3.6389)
2016.year	0.0000 (.)	0.0000 (.)	0.0089 (0.0091)	2.2039 (4.9164)
_cons	0.2325 *** (0.0549)	78.1065 *** (6.5723)	-0.0054 (0.0076)	56.3153 *** (2.7143)
*N*	9986	2360	9986	1630

DID: Difference-in-Differences, OOP: Out-of-pocket, GLM: Generalized linear model Standard errors in parentheses ^*^ *p* <0.05, ^**^ *p* <0.01, ^***^ *p* <0.001

With time-demeaned data, however, the coefficient on the interaction term (policy × post) becomes negative − 0.0153 (p =0.490) in the first part and − 7.4494 (p =0. 018) in the second part, respectively, indicating that with the time demeaned data both the probability to spend and the amount of spending decrease after the policy compared to those of the control group even if only the coefficient of the second part is statistically significantly associated with the policy.

The marginal effects on interaction term (policy × post) displays − 470102.4, suggesting that the policy group’s inpatient OOP payments decrease by USD 358.86 after policy compared to those of the control group, which shows a statistically and practically significant association.

***Out-of-pocket payments for outpatient services*.** For outpatient services, the coefficient on the interaction term (policy × post) is − 0.0070 (p =0.058) in the first part, implying that the probability of the policy group spending outpatient OOP payments decreases after policy compared to those of the control group, which is statistically insignificant associated with the policy. Conditional on patients with any outpatient spending, the coefficient on the interaction term (policy × post) appears − 123.44 (p =0.000) in the second part, suggesting that the policy group’s spending for outpatient services decreased after the policy compared to those of the control group, which shows statistically significant association with the policy ( Table 6).

**Table 6.** Coefficients from the DID model for outpatients OOP payments.

	GLM		GLM(w/time-demeaned data)	
	First part	Second part	First part	Second part
Policy	0.0019 (0.0023)	76.5264 ** (24.8509)	0.0046 (0.0032)	-183.5800 * (88.6045)
Post	-0.0009 (0.0043)	-16.0668 (48.8532)	0.0082 (0.0043)	-222.7847 (145.0996)
Policy#Post	-0.0070 (0.0037)	-123.4373 *** (33.9255)	-0.0067 * (0.0034)	57.5946 (111.9957)
Age	-0.0000 (0.0001)	-0.4263 (1.0705)	-0.0019 (0.0010)	8.3548 (32.8569)
Female	0.0035 (0.0021)	5.3401 (19.9360)	0.0000 (.)	0.0000 (.)
Edu_high school	-0.0023 (0.0020)	-55.5804 * (22.5301)	-0.0006 (0.0017)	-23.2082 (287.5015)
Edu_College	-0.0014 (0.0033)	21.5929 (27.6988)	-0.0010 (0.0019)	226.1822 (170.7289)
Married	0.0060 * (0.0029)	34.4097 (23.2584)	0.0158 (0.0108)	16.9181 (90.9824)
Children	0.0005 (0.0011)	-17.4761 (13.4977)	-0.0011 (0.0022)	17.3038 (60.7652)
Seoul	-0.0011 (0.0034)	52.0227 * (22.9793)	0.0024 (0.0063)	-137.4080 (190.6667)
House income	0.0000 (0.0000)	0.0145 *** (0.0032)	0.0000 (0.0000)	0.0193 (0.0102)
Employed	0.0030 (0.0030)	81.1650 * (35.5722)	0.0044 (0.0026)	-3.5043 (43.6498)
Medical Expense	0.0000 (0.0000)	0.0001 *** (0.0000)	0.0000 * (0.0000)	0.0001 *** (0.0000)
Individual Income	-0.0000 (0.0000)	-0.0056 (0.0060)	-0.0000 (0.0000)	-0.0495 (0.0415)
Number of Chronic Disease	0.0005 (0.0005)	28.0394 *** (4.8110)	-0.0001 (0.0005)	30.6983 * (14.1805)
Perceived Health_Very good	0.0016 (0.0049)	-52.1225 (40.5430)	0.0052 (0.0072)	10.7111 (94.8293)
Good	-0.0009 (0.0019)	-34.6203 (20.4976)	-0.0011 (0.0019)	1.7752 (39.6369)
Bad	0.0019 (0.0019)	24.2418 (23.0237)	0.0067 ** (0.0026)	39.1585 (63.6432)
Very Bad	-0.0010 (0.0069)	-53.3244 (60.6735)	0.0017 (0.0091)	-118.4526 (187.4564)
BMI	0.0006 (0.0003)	1.8492 (2.4760)	0.0016 * (0.0007)	-5.2781 (4.8080)
Disabled	-0.0005 (0.0031)	-21.8775 (28.1514)	-0.0038 (0.0101)	302.7723 * (151.6629)
PHI (Private Health Insurance)	0.0003 (0.0027)	51.5572 (27.6609)	-0.0058 (0.0047)	122.2981 (122.1676)
Total_Premium for PHI	-0.0000 (0.0000)	-0.0000 (0.0001)	-0.0000 (0.0000)	0.0003 (0.0003)
Number of PHI	0.0013 * (0.0006)	-0.8503 (11.5480)	0.0058 (0.0036)	-70.4579 (44.6626)
2010.year	-0.0008 (0.0030)	23.8529 (31.6741)	0.0006 (0.0027)	31.0881 (67.4938)
2011.year	0.0028 (0.0026)	63.8545 * (31.6837)	0.0056 * (0.0022)	16.7748 (80.3111)
2012.year	0.0000 (0.0031)	66.4134 (34.3596)	0.0040 (0.0028)	16.8925 (102.4408)
2013.year	0.0001 (0.0036)	174.2582 *** (42.3157)	-0.0022 (0.0028)	238.1435 * (98.3367)
2014.year	-0.0023 (0.0029)	-50.6203 (30.7784)	0.0019 (0.0018)	-20.5294 (69.6188)
2015.year	0.0013 (0.0025)	-57.6566 (35.6834)	0.0043 * (0.0020)	-45.1179 (79.9959)
2016.year	0.0000 (.)	0.0000 (.)	0.0032 (0.0019)	95.6490 (84.0325)
_cons	0.9700 *** (0.0122)	317.1954 ** (109.0522)	-0.0026 (0.0015)	542.9986 *** (62.2696)
*N*	9986	9929	9986	3619

DID: Difference-in-Differences, OOP: Out-of-pocket, GLM: Generalized linear model Standard errors in parentheses ^*^ *p* <0.05, ^**^ *p* <0.01, ^***^ *p* <0.001

With the time demeaned data, the coefficient on the interaction term (policy × post) in the first part is still negative, − 0.0067 (p =0.049), but the coefficient on the interaction term (policy × post) in the second part becomes positive, 57.59 (p =0.607). However, it is statistically insignificant associated with the policy.

**Figure 1.**
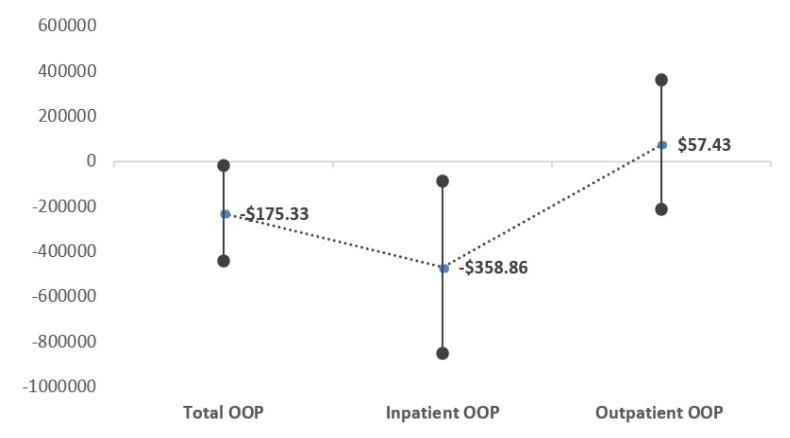
Marginal effects of the policy group after policy change. After the policy change, the policy group's out-of-pocket payments decreased by USD 175.33 (total) and USD 358.86 (inpatient services), respectively, which are statistically significant. In the case of out-patient services, patients’ health spending increased by USD 57.45, which is statistically insignificant.

The marginal effect on the interaction term (policy × post) shows 75233.4, indicating that the policy group’s OOP payments for outpatient services increase by USD 57.43 after policy compared to those of the control group, which is statistically insignificantly associated with the policy ( Figure 1).

### Utilizations

***Hospital days for inpatient services*.** For hospital days, the coefficient on the interaction terms (policy × post) displays 0.016 (p =0.376) in the first part and 0.0996 (p-value =0.368) in the second part conditional on patients with any day in hospitals. The results suggest that not only the probability of the policy group’s hospitalizing but also the hospital days increased after the policy compared to those of the control group even though both coefficients are statistically insignificantly associated with the policy ( Table 7).

**Table 7.** Coefficients from the DID model for stays in hospitals.

	GLM		GLM(w/time-demeaned data)	
	First part	Second part	First part	Second part
Policy	0.0874 *** (0.0144)	-0.1226 (0.0907)	0.1298 *** (0.0228)	-0.0101 (0.1486)
Post	0.0520 * (0.0221)	-0.1920 (0.1558)	0.0309 (0.0220)	0.3055 (0.4557)
Policy#Post	0.0160 (0.0181)	0.0996 (0.1107)	-0.0091 (0.0226)	0.0776 (0.1880)
Age	-0.0004 (0.0005)	-0.0039 (0.0031)	-0.0034 (0.0053)	-0.0516 (0.1003)
Female	-0.0244 * (0.0097)	-0.1986 ** (0.0623)	0.0000 (.)	0.0000 (.)
Edu_high school	0.0024 (0.0101)	-0.2271 *** (0.0639)	0.0713 (0.0896)	0.4757 (0.4295)
Edu_College	-0.0228 (0.0122)	-0.1154 (0.0862)	-0.0060 (0.0719)	0.1277 (0.8317)
Married	-0.0133 (0.0111)	-0.2021 ** (0.0705)	0.0315 (0.0448)	-0.0243 (0.3250)
Children	0.0009 (0.0046)	0.0201 (0.0295)	0.0120 (0.0123)	0.0329 (0.0988)
Seoul	-0.0511 *** (0.0115)	-0.2859 ** (0.0918)	0.1277 ** (0.0478)	-0.7469 ** (0.2850)
House income	-0.0000 *** (0.0000)	-0.0000 (0.0000)	-0.0000 *** (0.0000)	0.0000 (0.0000)
Employed	-0.0201 (0.0134)	-0.2452 *** (0.0732)	0.0131 (0.0129)	-0.1253 (0.1096)
Medical Expense	0.0000 *** (0.0000)	0.0000 *** (0.0000)	0.0000 *** (0.0000)	0.0000 *** (0.0000)
Individual Income	-0.0000 (0.0000)	-0.0001 ** (0.0000)	0.0000 * (0.0000)	-0.0001 (0.0001)
Number of Chronic Disease	-0.0061 * (0.0024)	-0.0356 * (0.0147)	-0.0032 (0.0032)	-0.0028 (0.0352)
Perceived Health_Very good	0.0039 (0.0247)	-0.3488 ** (0.1337)	0.0190 (0.0282)	-0.4866 * (0.2439)
Good	0.0098 (0.0097)	-0.0621 (0.0611)	0.0062 (0.0112)	-0.0487 (0.1000)
Bad	0.0386 *** (0.0113)	0.2100 ** (0.0655)	0.0205 (0.0131)	0.0507 (0.1060)
Very Bad	0.0793 * (0.0371)	0.6485 *** (0.1426)	0.0573 (0.0387)	0.4782 * (0.2393)
BMI	-0.0030 * (0.0015)	-0.0125 ** (0.0046)	-0.0045 ** (0.0016)	-0.0329 ** (0.0126)
Disabled	0.0261 (0.0172)	0.2375 * (0.0985)	-0.0399 (0.0387)	-0.3476 (0.2826)
PHI (Private Health Insurance)	0.0145 (0.0123)	-0.0434 (0.0865)	0.0336 (0.0268)	-0.5014 * (0.2443)
Total_Premium for PHI	0.0000 (0.0000)	-0.0000 (0.0000)	0.0000 (0.0000)	-0.0000 (0.0000)
Number of PHI	-0.0057 (0.0043)	0.0580 * (0.0268)	-0.0220 (0.0115)	0.0559 (0.0989)
2010.year	-0.0006 (0.0151)	-0.0366 (0.1311)	0.0028 (0.0127)	0.1261 (0.1889)
2011.year	-0.0100 (0.0153)	-0.1740 (0.1247)	-0.0015 (0.0119)	-0.0119 (0.2205)
2012.year	0.0056 (0.0164)	-0.2971 * (0.1285)	-0.0022 (0.0135)	-0.1000 (0.3176)
2013.year	-0.0380 (0.0201)	-0.1623 (0.1303)	0.0061 (0.0155)	-0.4360 (0.2369)
2014.year	-0.0214 (0.0141)	0.0304 (0.0858)	-0.0010 (0.0103)	-0.4303 * (0.1726)
2015.year	-0.0181 (0.0147)	0.0586 (0.0848)	0.0063 (0.0103)	-0.3517 (0.1948)
2016.year	0.0000 (.)	0.0000 (.)	0.0080 (0.0092)	-0.3981 (0.2670)
_cons	0.2557 *** (0.0549)	3.6406 *** (0.2890)	-0.0028 (0.0078)	2.2686 *** (0.1700)
*N*	9986	2444	9986	1658

DID: Difference-in-Differences, GLM: Generalized linear model Standard errors in parentheses ^*^ *p* <0.05, ^**^ *p* <0.01, ^***^ *p* <0.001

With the time-invariant data, the coefficient on the interaction term (policy × post) becomes negative − 0.009 (p =0.689) in the first part, but still positive 0.078 (p =0.680) in the second part, indicating that the policy group’s probability of hospitalizing decrease after policy, but conditional on patients with any day in hospitals, hospital days of the policy group increase compared to those of the control group even if both coefficients are statistically insignificantly associated with the policy.

As the marginal effect of the hospital days presents 0.9, it implies that the policy group’s hospital days increase on average 0.9 days more than the control group after the policy even though it is statistically insignificantly associated with the policy. 

***Number of visits for outpatient services*.** For the number of visits, the coefficient on the interaction term (policy × post) indicates − 0.003 (p =0.059) in the first part and − 0.018 (p =0.683) in the second part, suggesting that the policy group is less likely to visit compared to the control group after policy. The number of visits for outpatient services also decreases compared to the control group even though both coefficients are statistically insignificantly associated with the policy ( Table 8).

**Table 8.** Coefficients from the DID model for visits to hospitals.

	GLM		GLM(w/time- demeaned data)	
	First part	Second part	First part	Second part
Policy	0.0014 * (0.0006)	-0.1527 *** (0.0365)	0.0016 (0.0010)	0.2247 * (0.1057)
Post	-0.0014 (0.0011)	-0.5024 *** (0.0521)	0.0023 (0.0022)	-0.1972 (0.2798)
Policy#Post	-0.0033 (0.0017)	-0.0181 (0.0443)	0.0002 (0.0015)	-0.1764 (0.1099)
Age	0.0000 (0.0001)	0.0157 *** (0.0016)	-0.0007 (0.0005)	0.0968 (0.0607)
Female	0.0015 (0.0009)	0.1211 *** (0.0288)	0.0000 (.)	0.0000 (.)
Edu_High school	-0.0002 (0.0005)	-0.0542 (0.0301)	0.0003 (0.0009)	-0.2408 (0.2299)
Edu_College	-0.0017 (0.0018)	-0.1069 ** (0.0347)	-0.0014 (0.0014)	0.2115 (0.4182)
Married	0.0012 (0.0012)	-0.0357 (0.0341)	0.0055 (0.0084)	0.0818 (0.2035)
Children	0.0001 (0.0003)	-0.0114 (0.0157)	-0.0021 (0.0014)	-0.0769 (0.0684)
Seoul	0.0003 (0.0010)	-0.0152 (0.0392)	-0.0019 (0.0016)	-0.4483 (0.3262)
House income	0.0000 (0.0000)	-0.0000 (0.0000)	0.0000 (0.0000)	0.0000 (0.0000)
Employed	0.0060 * (0.0024)	-0.0184 (0.0313)	0.0032 (0.0020)	0.2591 ** (0.0793)
Medical Expense	-0.0000 (0.0000)	0.0000 *** (0.0000)	-0.0000 (0.0000)	0.0000 *** (0.0000)
Individual Income	0.0000 (0.0000)	-0.0000 (0.0000)	-0.0000 (0.0000)	-0.0001 * (0.0000)
Number of Chronic Disease	0.0001 (0.0001)	0.0995 *** (0.0062)	-0.0001 (0.0002)	0.0286 (0.0215)
Perceived Health_Very good	0.0020 ** (0.0008)	-0.1629 *** (0.0485)	0.0004 (0.0005)	-0.3467 * (0.1404)
Good	0.0014 (0.0008)	-0.0506 * (0.0218)	0.0012 * (0.0005)	-0.0182 (0.0619)
Bad	0.0026 ** (0.0009)	0.1338 *** (0.0252)	0.0049 ** (0.0015)	0.0627 (0.0612)
Very Bad	0.0036 * (0.0014)	0.1694 * (0.0704)	0.0070 * (0.0031)	0.1061 (0.1573)
BMI	0.0006 * (0.0003)	0.0085 ** (0.0032)	0.0017 ** (0.0006)	0.0028 (0.0090)
Disabled	-0.0031 (0.0022)	0.0348 (0.0457)	-0.0068 (0.0053)	-0.1735 (0.1918)
PHI (Private Health Insurance)	0.0010 (0.0009)	-0.0237 (0.0364)	-0.0028 (0.0039)	-0.0549 (0.1480)
Total_Premium for PHI	-0.0000 (0.0000)	-0.0000 (0.0000)	-0.0000 (0.0000)	-0.0000 (0.0000)
Number of PHI	-0.0002 (0.0003)	0.0022 (0.0114)	0.0029 (0.0031)	0.0571 (0.0606)
2010.year	-0.0022 * (0.0010)	0.0479 (0.0299)	-0.0012 (0.0009)	0.1996 (0.1134)
2011.year	-0.0012 ** (0.0004)	0.0719 * (0.0313)	0.0012 (0.0011)	0.0477 (0.1389)
2012.year	-0.0021 (0.0012)	0.0889 ** (0.0343)	0.0003 (0.0013)	0.0814 (0.1843)
2013.year	0.0003 (0.0011)	0.6055 *** (0.0445)	-0.0008 (0.0009)	0.2900 * (0.1456)
2014.year	-0.0025 (0.0016)	-0.0127 (0.0259)	-0.0005 (0.0007)	-0.0757 (0.1026)
2015.year	-0.0007 (0.0013)	-0.0075 (0.0226)	0.0005 (0.0010)	-0.1963 (0.1239)
2016.year	0.0000 (.)	0.0000 (.)	0.0010 (0.0009)	-0.1260 (0.1605)
_cons	0.9768 *** (0.0086)	1.7594 *** (0.1502)	0.0000 (0.0006)	2.2571 *** (0.0997)
*N*	9986	9975	9986	4128

DID: Difference-in-Differences, GLM: Generalized linear model Standard errors in parentheses ^*^ *p* < 0.05, ^**^ *p* < 0.01, ^***^ *p* < 0.001

With time-demeaned data, however, the coefficient on the interaction term (policy × post) becomes positive 0.0002 (p =0.920) in the first part and negative − 0.1764 (p =0.108) in the second part, implying that the policy group is more likely to visit after the policy than the control group. However, the policy group’s number of visits decreased compared to the control group after the policy. Both coefficients are statistically insignificantly associated with the policy.

**Figure 2.**
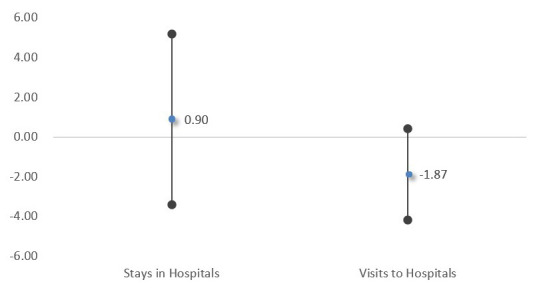
Marginal effects of the policy group for utilization. There were no statistically significant changes after the policy change in term of patients' utilization for both inpatient and outpatient services.

The marginal effect of the interaction term (policy × post) for outpatient services shows − 1.87 in the second part which is suggested that the policy group’s visits decreased by 1.87 after the policy compared to the control group ( Figure 2).

### Triple differences (TD) by patients’ income levels

As shown in Table 9, the coefficient on the interaction term (policy × post) is − 0.002 (p =0.796) in the first part, implying that the probability of the highest income group’s (reference group) spending OOP payment decreases compared to patients in other income levels after the policy change even though it is statistically insignificantly associated with the policy.

**Table 9.** Coefficients from the triple differences.

	GLM		GLM(w/time- demeaned data)	
	First part	Second part	First part	Second part
Policy	-0.0004 (0.0034)	0.4305 *** (0.0996)	0.0003 (0.0041)	0.0164 (0.4614)
Post	-0.0037 (0.0058)	0.2033 (0.1404)	0.0047 (0.0042)	0.5453 (0.9488)
House Income(1)	0.0018 (0.0034)	0.0371 (0.1105)	-0.0023 (0.0021)	-0.6056 (0.4178)
House Income(2)	0.0005 (0.0038)	-0.1054 (0.1260)	-0.0086 ** (0.0032)	-1.2026 (0.6158)
House Income(3)	-0.0021 (0.0044)	-0.2879 (0.1545)	-0.0129 ** (0.0040)	-1.8366 (0.9706)
Policy#Post	-0.0018 (0.0071)	-0.8680 ** (0.3014)	-0.0029 (0.0038)	-2.5386 *** (0.4281)
Post#House Income(1)	0.0018 (0.0050)	-0.3181 * (0.1425)	-0.0007 (0.0061)	0.3424 (0.5514)
Post#House Income(2)	0.0050 (0.0049)	-0.0078 (0.1353)	0.0020 (0.0070)	1.3760 * (0.6542)
Post#House Income(3)	0.0111 * (0.0052)	0.0961 (0.1595)	0.0069 (0.0063)	1.9498 * (0.7953)
Policy#House Income(1)	-0.0033 (0.0056)	-0.0424 (0.1338)	-0.0024 (0.0039)	-2.2739 *** (0.4511)
Policy#House Income(2)	0.0010 (0.0057)	0.1356 (0.1382)	0.0028 (0.0032)	-0.0087 (0.6984)
Policy#House Income(3)	0.0081 (0.0047)	0.1612 (0.1610)	0.0054 (0.0040)	1.0210 (0.5519)
Policy#Post# House Income(1)	-0.0004 (0.0092)	0.8167 ** (0.3127)	-0.0066 (0.0085)	3.5973 *** (0.7472)
Policy#Post# House Income(2)	-0.0058 (0.0101)	0.2637 (0.3333)	-0.0021 (0.0081)	1.0715 (0.8105)
Policy#Post# House Income(3)	-0.0094 (0.0087)	0.5850 (0.3285)	-0.0025 (0.0071)	0.8418 (0.8375)
Age	-0.0001 (0.0001)	-0.0029 (0.0040)	-0.0013 (0.0009)	-0.1260 (0.2237)
Female	0.0029 (0.0021)	-0.0773 (0.0623)	0.0000 (.)	0.0000 (.)
Edu_high school	-0.0019 (0.0019)	-0.1469 * (0.0630)	-0.0030 (0.0022)	0.6962 (7.1447)
Edu_college	-0.0007 (0.0032)	0.0616 (0.0771)	0.0010 (0.0015)	1.5834 (1.4777)
Married	0.0066 * (0.0030)	0.0357 (0.0940)	0.0080 (0.0076)	2.4681 *** (0.3277)
Children	0.0008 (0.0012)	-0.0699 (0.0389)	0.0001 (0.0017)	0.0898 (0.3546)
Seoul	-0.0007 (0.0032)	0.0501 (0.0606)	0.0031 (0.0061)	0.8956 (0.6987)
Employed	-0.0020 (0.0020)	0.1040 (0.1248)	0.0007 (0.0020)	-0.3311 (0.2629)
Medical Expense	0.0000 *** (0.0000)	0.0000 *** (0.0000)	0.0000 *** (0.0000)	0.0000 *** (0.0000)
Individual Income	0.0000 (0.0000)	0.0000 (0.0000)	-0.0000 (0.0000)	0.0001 (0.0001)
Number of Chronic Diseases	0.0004 (0.0004)	-0.0002 (0.0126)	-0.0001 (0.0005)	0.0077 (0.0946)
Perceived Health_ Very good	0.0006 (0.0049)	-0.2790 (0.1445)	0.0049 (0.0071)	-0.7100 (0.4625)
Good	-0.0014 (0.0018)	-0.3761 * (0.1794)	-0.0022 (0.0019)	-0.1711 (0.2377)
Bad	-0.0002 (0.0017)	0.0801 (0.0595)	0.0033 (0.0023)	-0.0016 (0.1965)
Very Bad	-0.0044 (0.0067)	0.3863 *** (0.0863)	-0.0032 (0.0087)	0.4593 (0.4413)
BMI	0.0001 (0.0002)	0.0000 (0.0049)	0.0003 (0.0003)	-0.0051 (0.0257)
Disabled	0.0029 (0.0022)	0.0278 (0.0827)	0.0005 (0.0089)	-1.0627 (0.6369)
PHI(Private Health Insurance)	-0.0005 (0.0026)	0.2648 ** (0.0808)	-0.0038 (0.0030)	1.0670 ** (0.3979)
Total_Premium for PHI	-0.0000 (0.0000)	-0.0000 (0.0000)	-0.0000 (0.0000)	-0.0000 (0.0000)
Number of PHI	0.0013 * (0.0006)	-0.0435 (0.0305)	0.0029 (0.0019)	-0.0744 (0.1031)
2010.year	0.0009 (0.0028)	0.0034 (0.0830)	0.0015 (0.0025)	-0.0178 (0.3198)
2011.year	0.0034 (0.0025)	0.0690 (0.0781)	0.0041 * (0.0020)	0.5207 (0.4342)
2012.year	0.0014 (0.0027)	0.1348 (0.0750)	0.0035 (0.0026)	0.0565 (0.6353)
2013.year	0.0002 (0.0036)	0.2814 * (0.1144)	-0.0018 (0.0027)	0.6279 (0.4374)
2014.year	-0.0009 (0.0027)	-0.0532 (0.1485)	0.0018 (0.0017)	0.1995 (0.3517)
2015.year	0.0017 (0.0024)	-0.0279 (0.1230)	0.0034 (0.0018)	-0.1609 (0.3734)
2016.year	0.0000 (.)	0.0000 (.)	0.0025 (0.0017)	0.1037 (0.4132)
_cons	0.9875 *** (0.0107)	13.7239 *** (0.3229)	-0.0022 (0.0014)	11.7664 *** (0.3417)
*N*	10009	9959	10009	9959

GLM: Generalized linear model Standard errors in parentheses ^*^ *p* < 0.05, ^**^ *p* < 0.01, ^***^ *p* < 0.001 For triple interaction (policy × post × income level), income levels are categorized by four different income groups, from high to low, specifying 'House Income (0)' is the reference group, the highest income level. 'House Income (1)' = the second highest income level, 'House Income (2)' = the third highest income level, and 'House Income (3)' = the lowest income level.

As all coefficients on the triple interaction terms (policy × post × hhinc_c) are negative, −0.0004 (p =0.967), − 0.0058 (p =0.564), − 0.0094 (p =0.281), respectively, in the first part, suggesting that the probability of spending OOP payments decreases in all income levels compared to the reference group even though they are statistically insignificantly associated with the policy.

In the second part, conditional on patients with any spending for OOP payments, the coefficient on the interaction term (policy × post, reference group, the highest income group) is −0.8680 (p =0.004), indicating that the amount of total OOP payments spent by the highest income group decreases after the policy, statistically significantly associated with the policy. However, the coefficients on other triple interaction terms (policy × post × hhinc_c) are all positive values (0.8167, 0.2637, 0.5850, respectively), indicating that OOP payments are most reduced in the highest income level (reference group). The other income levels are less decreased than the highest income level. Our analysis suggests that the benefits expansion policy does not affect the patients' health spending by their income levels.

With the time-demeaned data, the coefficient on the interaction (policy × post, reference group, the highest income group) is − 0.0029 (p =0.439). The coefficients on the triple interaction terms (policy × post × hhinc_c) are all negative (− 0.007 [p =0.437], − 0.002 [p =0.791], − 0.025 [p =0.724]) in the first part as shown in Table 8. In all income levels, the probability of spending decreased after the policy change compared to the reference group (the highest income level) although they are all statistically insignificantly associated with the policy.

In the second part, the coefficient on the interaction terms (policy × post) is − 2.539 (p =0.000) and the coefficients on the triple interaction terms (policy × post × hhinc_c) are all positive (3.5973, 1.0715, 0.8418), indicating that the OOP payments are most reduced in the highest income group. However, with the time-demeaned data, the magnitude of the triple interaction terms is the highest in the lowest income group, − 1.6968 (− 2.5386 + 0.8418), suggesting that OOP payments are most reduced in the lowest income group after the policy except for the reference group, followed by the third highest income group, − 1.4671 (− 2.5386 + 1.0715) and the second highest income group, 1.0587 (− 2.5386 + 3.5873).

That is, with the time-demeaned data, the effects of the policy are somehow in accordance with the income levels. However, the OOP payments are still most decreased in the highest income levels, which are statistically significantly associated with the policy.

**Figure 3.**
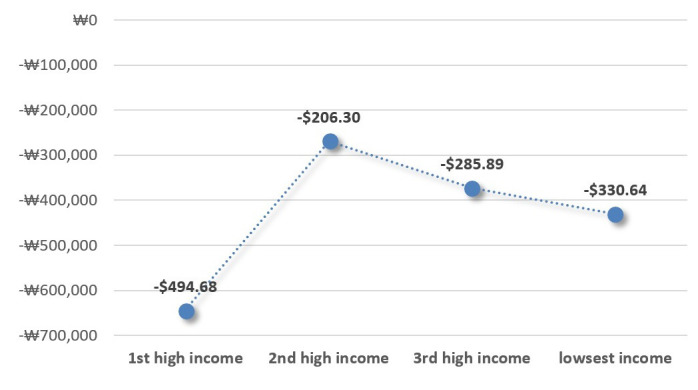
The patients’ expected reduction in OOP payments by income groups The out-of-pocket payments were most reduced at the highest income level (USD 494.68) after the policy implementation, followed by the lowest income (USD 330.64), the third-highest income level (USD 285.89), and the second-highest income level (USD 206.30), respectively.

The marginal effect of the reference group (the highest income group) is – USD 494.68. The marginal effects of the triple interaction terms (policy × post × hhinc_c) are – USD 206.30 in the second-highest income, − USD 285.89 in the third-highest income, and – USD 330.64 in the lowest income, respectively ( Figure 3).

From the Triple Differences estimation, we can identify that the benefits expansion policy does not proportionally affect the patients’ health expenditure by their income levels. The OOP payments are most reduced at the highest income level. This result corresponds to Kim and Kwon’s (2015) research that catastrophic payments decrease more in the high-income group than in the low-income group after the benefits expansion policy in South Korea.

## Discussion

 This study examines the effects of the benefit expansion policy on patients’ health expenditure and utilization in South Korea. As of 2013, the government endeavored to reduce the patients’ financial burden for healthcare, mainly for patients with major catastrophic diseases, by increasing the portion of covered services in a package. Our findings indicate that the policy group’s total and inpatient OOP payments decreased by USD 175.33 and USD 358.86, respectively, of which both coefficients were statistically significantly associated with the policy. However, OOP payments for outpatient services statistically insignificantly increased by USD 57.43 after the policy.

 Regarding the patients’ utilization, the policy group’s hospital days for inpatient services increased by 0.9 days, and the number of visits for outpatient services decreased by 1.87. However, both coefficients appeared statistically insignificant associations with the policy.

This study finds that the benefits expansion policy affected to reduce the policy group’s total OOP payments and inpatient OOP payments but no significant changes in utilization. The patients’ health spending was largely reduced by inpatient services. This might result from the fact that the beneficiaries of the benefits expansion policy were substantially patients with major catastrophic diseases who necessarily needed inpatient services. This result supports that the rate of coverage expenditure for the for major catastrophic diseases was 81.7% in 2017, increased by 1.4% compared to the previous year ( Hwang *et al.*, 2021).

However, considering that the rate of coverage expenditure for other diseases, excluding the major catastrophic diseases, was 57.1% in 2017, decreased by 24.6% compared to the previous year ( Hwang *et al.*, 2021), some equity issues can arise between the patients.

In addition, we find that the OOP payments mostly decreased at the highest income level. This is because the volume of care consumed by high-income patients is generally larger than the lower income patients due to the discrepancy in financial capacity. Since the patients’ utilization did not significantly change after policy change, health spending of patients with high income could significantly decrease, as the costs of care were reduced by the policy. If the policy’s intention is primarily to reduce the lower-income patients’ health spending, this result suggests that the policy did not meet the goal.

In this study, we raise the estimate’s accuracy by carefully considering the features of the health claims data with adequate approaches such as the Two-Part Model and GLM model. By comparing the original GLM model to the GLM with the time-demeaned data, we also attempt to identify potentially omitted time-invariant individual effects.

Also, we embrace the lagged effects of the policy by using eight years of data, 2009 to 2012 [pre-periods] and 2013 to 2016 [post-periods]

 However, this study also has some limitations as well. The 'Mid-term Health Benefits Security Plan' has been implemented since 2005. Even though strategies for expanding benefits significantly changed in 2013, it is still the fact that initiatives have been implemented approximately for a decade. Thus, the effects of the policy in post-policy periods can be underestimated by an ongoing implementation of the policy.

Also, because of the difference in a recording system of the disease codes between the data set and the government’s guidelines, patients with relevant diseases that precisely were not supposed to be involved in the four major diseases might be inaccurately included in the policy group.

## Conclusion

This study aims to evaluate the effects of the government’s health benefits expansion policy. As a result, we find evidence that the policy reduced the patients’ health spending in terms of total OOP payments and inpatient OOP payments without significant changes in utilization. The OOP payments for inpatient services were primarily reduced as the main beneficiaries of the policy were patients with major catastrophic diseases who essentially needed inpatient services. We raise an equity issues as patients with other diseases were not able to take sufficient benefits from the policy.

Also, we find evidence that the policy’s impact significantly reduced the patients’ spending of the highest income level. Given that the purpose of the policy is to redistribute societal resources through the NIH, it suggests that the policy did not achieve its goal.

As the policy’s benefits were considerably limited to inpatient services and high-income patients, the benefits expansion policy needs to reinforce the range of covered services for the general population to be able to benefit from the policy.

## Ethics and consent

The use of data in this study was approved by the data archivist of the Korea Health Panel Study (KHPS). According to KHPS legislation, neither approval from the ethics committee nor informed consent from the study populations is required as this data is collected anonymously (which means that no identifiers can be connected to the data, either directly or through a coding system).

## Data Availability

The data for this study is owned by the Korea Health Panel Study (KHPS). The first panel study of 2009–2012 and 2013–2016 is used for the study and can be obtained here: https://www.khp.re.kr:444/web/data/data.do. The website in English is also available at https://www.khp.re.kr:444/eng/main.do. Data is publicly available upon permission from the authority. Therefore, users must download the ‘Korea Health Panel Survey Data User Agreement’ form and send it back to at khp@kihasa.re.kr to receive the data. For the control group, this study used the same data that we used for the policy group (Korea Health Panel Data), but we referred to the diseases names from the government’s report ‘2012 Annual Survey on Medical Expense of National Health Insurance Enrollees ( NHIC, 2013)’ and ‘Analysis on Patients of High-Paying and Critical Diseases among NHI Enrollees (2005)’ to sort out the control group from the data. Those reports list specific diseases causing high medical expenses per person. So, we referred to the names of the diseases to sort out the control group to make the control group comparably high with the policy group in terms of annual medical spending. We indicated 17 diseases in Table 1 that we selected to compose the control group.
