# Supplementary material for: Effects of national health benefits expansion policy on out-of-pocket payments and utilization of patients with four major catastrophic diseases
Source: Health Open Res. 2024 Feb 1;6:4. [Version 1] doi: 10.12688/healthopenres.13475.1 (PMC11638905; doi:10.12688/healthopenres.13475.1)
Supplement: Peer review history

# Reviewer report: Reviewer response for version 1

**DOI:** 10.21956/healthopenres.14562.r28769

**Published:** 2024-09-03

**Authors:** Christy Pu

**Affiliations:** 1 National Yang Ming Chiao Tung University, Taipei, Taiwan

This paper examines the effect of a Korean policy on out-of-pocket (OOP) expenses and healthcare utilization. The study addresses an important issue, as OOP costs in several countries with universal health coverage or national health insurance can still be significant and warrant careful government scrutiny. The paper is generally well-written, but I have a few suggestions:

1. The description of the Mid-term benefit policy should be elaborated further. For instance, who is eligible, and what are the associated costs? The introduction does not clearly specify which groups of people are being compared.
2. The definition of the case and control groups should be introduced early in the Methods section, including the criteria for their selection.
3. How did the author address sample attrition? It seems unlikely that all participants were followed for the full 8 years. Were those without complete follow-up data excluded?
4. How were the diagnoses determined? Were the diseases self-reported in the survey? How accurate are these reports?
5. Including a diagram to examine the parallel trend assumption would be beneficial.
6. If an estimate is statistically insignificant, discussing the size of the estimates is not particularly meaningful. The conclusion should indicate that there is no evidence of meaningful differences.
7. In the Discussion section, the discussion on the two-part model and lagged effects seems out of place. The justification for the regression methods should be included in the Methods section.

Is the work clearly and accurately presented and does it cite the current literature?

Yes

If applicable, is the statistical analysis and its interpretation appropriate?

Yes

Are all the source data underlying the results available to ensure full reproducibility?

Partly

Is the study design appropriate and is the work technically sound?

Yes

Are the conclusions drawn adequately supported by the results?

Yes

Are sufficient details of methods and analysis provided to allow replication by others?

Partly

Reviewer Expertise:

Out-of-pocket healthcare spending; Policy evaluation

I confirm that I have read this submission and believe that I have an appropriate level of expertise to confirm that it is of an acceptable scientific standard.

# Response

**Published:** 2024-09-04

**Authors:** Minjeong Kim

**Affiliations:** Oregon State University, Corvallis, Oregon, USA

Hi, I'm Minjeong Kim, the first author of the article. I sincerely appreciate your review. Thank you for your comments, I think all your comments are valuable and worthwhile. I'll look over my article again revise it reflecting your comments. Again, thank you so much your review and your time. Minjeong Kim

# Reviewer report: Reviewer response for version 1

**DOI:** 10.21956/healthopenres.14562.r28065

**Published:** 2024-05-13

**Authors:** Firdaus Hafidz

**Affiliations:** 1 Universitas Gadjah Mada, Yogyakarta, Indonesia

This research study evaluates the impact of the 2013 policy change on out-of-pocket (OOP) payments and healthcare service utilization in South Korea. The study is critical as it offers valuable insights for the development of health insurance policies in various countries, not just in Korea. 

 I found the article to be well-written; however, some clarifications and minor revisions are needed as outlined below

 In the "abstract background" section, it is mentioned that the expansion of health benefits has been ongoing since 2005, yet the data used spans from 2009 to 2016. It would be clearer to directly mention the policy changes in 2013 to provide context for the data range.

 Regarding the statement "if the range of the covered services is insufficient" – could you please elaborate on what the coverage was before and why it is considered to be insufficient? What are the most critical benefit packages needed that are currently not covered?

 For the time periods "(2009 to 2012 [pre], and 2013 to 2016 [post])", could you please clarify in the background what significant events or changes occurred before and after these years? Referring to President Park Gun-Hye's policy changes in the "Methods" section aids understanding, but moving this information to the "Background" would prevent redundancy and avoid duplication of the three-phase plan information.

 The term "the national health insurance enrollees" needs more context. If they are enrollees, is there a policy of out-of-pocket (OOP) costs? If so, does this relate to fee scheduling or cost sharing mentioned in the background?

 Could you confirm whether the unit of analysis in this study is the number of visits or individual patients with possibly multiple visits?

 Please provide information on the use of KCD-6 for the policy group classification .

 Regarding income grouping, was it based on quartiles of the Korean population or quartiles from the sample?

 In the "Methods" section, the explanation of income grouping is unnecessarily repeated. Please consolidate this information in one paragraph. Also, which year's income data was used? Does this imply that an individual's income status could change annually?

 Please correct the typo in the year noted in Table 2's footnotes: it currently reads "2009–201", which should be "2009–2012".

 In Table 3, please add whether the figures represent means or medians, and clarify whether the figures in parentheses are standard deviations (SD) or interquartile ranges (IQR).

 Considering the average age of the population is elderly for both the policy and control groups, could you clarify the inclusion and exclusion criteria of the study more distinctly? It appears that the group was only categorized based on the disease. Is this benefit specific to a particular subset of the population in the Korean context?

 Based on available data, the inflation rate in Korea from 2009 to 2016 was relatively stable. However, I would like to confirm if the inflation rate for healthcare was also stable, especially concerning payments to healthcare facilities, as this might affect the actual cost increases not leading to catastrophic expenses.

 In the "Discussion" section, it is mentioned that "If the policy’s intention is primarily to reduce lower-income patients’ health spending, this result suggests that the policy did not meet the goal." However, since the study does not include Medicaid as part of the poor, does this mean the policy's real intention was to protect the poor, or was it effectively protecting this group overall? Or what are the context of income in this group? 

 What policy and practical implications can you suggest based on these findings? This is relevant not only in Korea but also in other countries where expanded coverage is expected to reduce OOP costs and increase utilisation, but the impact has been different from expected.

 Lastly, I believe the conclusions are too bold in stating that the policy did not achieve its goals. Is it accurate to say that its intention of policy goal was to decrease inequity for this group?

 These comments aim to refine the manuscript and ensure that the analysis and implications are clearly understood and well-supported by the presented data.

Is the work clearly and accurately presented and does it cite the current literature?

Yes

If applicable, is the statistical analysis and its interpretation appropriate?

I cannot comment. A qualified statistician is required.

Are all the source data underlying the results available to ensure full reproducibility?

Partly

Is the study design appropriate and is the work technically sound?

Yes

Are the conclusions drawn adequately supported by the results?

Yes

Are sufficient details of methods and analysis provided to allow replication by others?

Yes

Reviewer Expertise:

I specialize in health financing and insurance, but I am not a mathematician equipped to verify the accuracy of the equations written. Additionally, due to time constraints, I am unable to examine all the statistical analysis tables in detail.

I confirm that I have read this submission and believe that I have an appropriate level of expertise to confirm that it is of an acceptable scientific standard.
